# COVID-19 Shuts Doors to Flu but Keeps Them Open to Rhinoviruses

**DOI:** 10.3390/biology10080733

**Published:** 2021-07-31

**Authors:** Irina Kiseleva, Andrey Ksenafontov

**Affiliations:** 1 Department of Virology, Institute of Experimental Medicine, 197376 Saint Petersburg, Russia; 2 Department of Etiology and Epidemiology, Smorodintsev Research Institute of Influenza, 197376 Saint Petersburg, Russia; ksenandrey@yandex.ru

**Keywords:** acute viral respiratory infections, pandemics, epidemics, influenza, SARS-CoV-2, COVID-19, influenza A virus, human rhinovirus

## Abstract

**Simple Summary:**

Ten years have passed since the beginning of the H1N1pdm09 flu pandemic. No sooner had humanity recovered from its consequences than a new attack came—the COVID-19 pandemic. What happens to other respiratory infectious diseases during a global disaster such as the COVID-19 pandemic? The pandemic brought about by the novel SARS-CoV-2 virus has disrupted many well-established epidemiological and pathogenetic relationships, as well as mechanisms affecting infections with other respiratory viruses. The level of circulation of many respiratory pathogens has changed significantly. For instance, global influenza activity is at much lower levels than expected. In many regions, the influenza season has not started. Intriguingly, the COVID-19 pandemic did not substantially affect the spread of human rhinoviruses. In this review, the main properties of epidemiologically significant respiratory viruses such as SARS-CoV-2, influenza virus, and human rhinovirus are described.

**Abstract:**

It is well known that rhinoviruses are distributed across the globe and are the most common cause of the common cold in all age groups. Rhinoviruses are widely considered to be harmless because they are generally perceived as respiratory viruses only capable of causing mild disease. However, they may also infect the lower respiratory tract, inducing chronic obstructive pulmonary disease and exacerbations of asthma, bronchiolitis, etc. The role of rhinoviruses in pathogenesis and the epidemiological process is underestimated, and they need to be intensively studied. In the light of recent data, it is now known that rhinoviruses could be one of the key epidemiological barriers that may influence the spread of influenza and novel coronaviruses. It has been reported that endemic human rhinoviruses delayed the development of the H1N1pdm09 influenza pandemic through viral interference. Moreover, human rhinoviruses have been suggested to block SARS-CoV-2 replication in the airways by triggering an interferon response. In this review, we summarized the main biological characteristics of genetically distinct viruses such as rhinoviruses, influenza viruses, and SARS-CoV-2 in an attempt to illuminate their main discrepancies and similarities. We hope that this comparative analysis will help us to better understand in which direction research in this area should move.

## 1. Introduction

Influenza and the common cold, also known simply as the cold, are acute viral infections of the upper respiratory tract. The common cold is known as one of the most ubiquitous infectious diseases in humans. In total, more than several hundred viral types are associated with acute viral respiratory infections. The most commonly implicated are human rhinoviruses (HRVs), which are the cause of more than half of all common colds [1]; human coronaviruses, accounting for 10–15% of colds; human respiratory syncytial viruses (HRSVs), also called human orthopneumoviruses, which are the most common cause of respiratory hospitalization in infants (up to 60%); influenza viruses; adenoviruses; human parainfluenza; and metapneumoviruses [1,2].

It is believed that viral colds are mostly self-limited infectious diseases that typically resolve within 8–10 days and are not serious illnesses [3]. However, the role of respiratory viruses—in particular, human rhinoviruses—in the total mass of respiratory pathogens cannot be underestimated. Rhinoviruses are ubiquitous respiratory tract pathogens. They affect the respiratory tract from its upper to lower parts, causing common colds, but have also been associated with pneumonia, asthma exacerbations, and wheezing [4]. In most cases, rhinoviruses as typical common cold pathogens lead to a short self-limiting illness. Most patients infected with HRVs are asymptomatic or suffer only mild symptoms [5]. However, for immunocompromised patients, the elderly, and asthmatics, HRV infections can lead to critical complications [6].

In addition, rhinoviruses are unique pathogens whose circulation is not affected by cataclysms such as epidemics and pandemics. As over 100 serotypes of HRVs exist, little immunological protection is provided to humans by prior rhinovirus infection, which accounts for the high incidence of subsequent infections [6]. However, the significant number of serotypes does not explain why rhinoviruses did not disappear during pandemics. Many other respiratory viruses, such as human adenoviruses, also have a large number of serotypes [7], but this did not help them to survive in the COVID-19 pandemic.

At the beginning of the spread of SARS-CoV-2 around the world, it was suggested that the pandemic would likely develop in accordance with one of two scenarios: (i) SARS-CoV-2 viruses would co-circulate with other viral respiratory pathogens, or (ii) SARS-CoV-2 viruses would displace them [8]. However, as the pandemic developed, it became clear that a third, combined scenario might have taken place. It was reported that the co-circulation of different respiratory viruses with SARS-CoV-2 strains could lead to both cooperative (rhinoviruses) and competitive (influenza viruses) forms of virus–virus interactions.

What happened to rhinoviruses in the COVID-19 era? What happened to other respiratory viruses in the COVID-19 era? What allowed two viruses—SARS-CoV-2 and rhinoviruses—to coexist peacefully, while SARS-CoV-2 displaced the influenza virus? In order to attempt to understand what unites these three respiratory viruses (SARS-CoV-2, influenza A virus, and human rhinovirus) and what the differences between them are, below we compared them according to several main characteristics. The results of this search are summarized in Table 1, Table 2 and Table 3.

## 2. Virus Taxonomy

**Figure 1:**
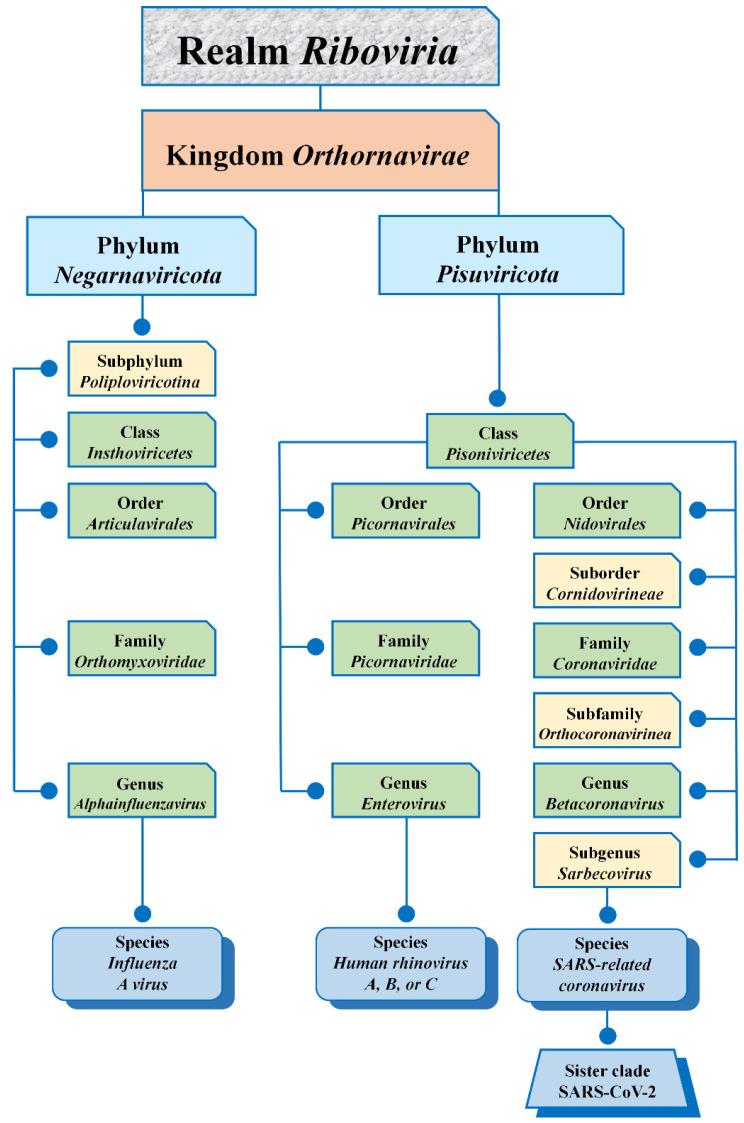
Taxonomic information on the three respiratory viruses discussed in this review [7,9].

All three viruses belong to the kingdom *Orthornavirae* [7]. One of them, influenza A virus (IAV), belongs to the phylum *Negarnaviricota*, and two others, HRVs and SARS-CoV-2, are members of the class *Pisoniviricetes*, phylum *Pisuviricota* (Figure 1). Based on phylogeny, taxonomy, and established practice, the International Committee on Taxonomy of Viruses recognizes SARS-CoV-2 as forming a sister clade to the prototype of the species SARS-related coronavirus [9]. To date, HRVs continue to co-circulate together with another representative of class *Pisoniviricetes*, SARS-CoV-2 [10]. In contrast, representatives of the phylum *Negarnaviricota*, human influenza viruses, demonstrated a mysterious phenomenon of the displacement of previously circulating influenza viruses by a new pandemic of SARS-CoV-2 [11,12,13].

What distinguishes the representatives of these two phyla? Why do some circulate in the COVID-19 pandemic while others do not?

## 3. Virion Morphology

Fully developed infectious viral particles of IAV, SARS-CoV-2, and HRV are composed of RNA surrounded by a protein coating. All these viruses assemble an icosahedral capsid with a cubical symmetry type (Table 1).

By conventional negative-staining electron microscopy, virions of SARS-CoV-2 and influenza A virus appear to be roughly spherical or pleiomorphic and medium-sized in diameter; surface projections are comprised of spike glycoproteins. In contrast, human rhinoviruses have smaller sizes [14].

**Table 1:** Comparative characteristics of HRV, IAV, and SARS-CoV-2: organization and replication.

Properties [refs]	Causative Agent		
	Rhinovirus	Influenza a Virus	SARS-CoV-2
VIRION MORPHOLOGY [15,16,17,18,19,20,21]			
Size (nucleocapsid diameter)	Small-sized (~30 nm)	Medium-sized (80–120 nm)	Medium-sized (~120 nm)
Nucleocapsid symmetry	Icosahedral symmetry	Icosahedral symmetry	Icosahedral symmetry
Shape	Spherical shape	Spherical shape, sometimes filamentous morphology	Spherical shape
VIRION STRUCTURE [15,16,17,18,19,20,21,22,23,24,25,26,27,28,29,30,31,32,33]			
Capsid (coat protein)	Yes	Yes	Yes
Envelop	No	Yes	Yes
Glycoprotein spike	No	HA and NA	S glycoprotein
Polybasic cleavage site	No	Only in avian IAV	Yes
RNA dependent RNA polymerase	Yes	Yes	Yes
HA esterase glycoprotein	No	No	Yes
PHYSICOCHEMICAL PROPERTIES [2,33,34]			
Thermostability	Environmentally stable to temperature (heat-resistant)	Environmentally labile to temperature (heat-sensitive)	Environmentally labile to temperature (heat-sensitive)
Stability to drying	Stable to drying	Disrupted by drying	Disrupted by drying
Stability to detergents	Stable to detergents	Disrupted by detergents	Disrupted by detergents
Acid stability, pH sensitivity	Unstable below pH 5–6	Disrupted by acid	Disrupted by acid
GENOME ORGANIZATION [22,26,27]			
Nucleic acid structure	RNA	RNA	RNA
Size	~7 kb	~14 kb	~30 kb
Type of nucleic acid molecule	Linear	Linear	Linear
Helix form of nucleic acid	Single-stranded	Single-stranded	Single-stranded
Segmented/nonsegmented genome	Nonsegmented	Segmented	Nonsegmented
Positive/negative-strand RNA	Positive-sense RNA	Negative-sense RNA	Positive-sense RNA
VIRAL REPLICATION [2,22,29,35,36,37,38,39,40,41]			
Recognition and attachment	A canyon in VP1 protein is the site of the attachment of sialic acids to the cell surface	Binding to sialic acids on a host cell by the receptor-binding domain of HA1	Binding to the cell surface protein ACE2 by S protein
Entry into the host-cell	Endocytosis, pinocytosis	Endocytosis	Endocytosis or at plasma membrane fusion
Infectivity of naked genomic RNA	Infectious	Not infectious	Infectious
Transcription and replication	Cytoplasm	Nucleus	Cytoplasm
Synthesis of viral proteins	Cytoplasm	Cytoplasm	Cytoplasm
Virion assembly	Cytoplasm	Cytoplasm	Cytoplasm
Virion release	Lytic or non-lytic releases	Budding	Exocytosis

## 4. Virion Structure

All viruses are categorized into two types based on their outer structure: (**i**) enveloped and (**ii**) non-enveloped [31]. IAV and SARS-CoV-2 are known to be lipid-enveloped viruses. The virion envelope of IAV and SARS-CoV-2 is derived from the cell membrane, incorporating virus glycoproteins and non-glycosylated proteins.

The lipid envelope of SARS-CoV-2 contains an envelope protein, a spike protein S, and a membrane protein. The nucleocapsid protein forms the virion core encased in the viral genome [17]. The S protein mediates viral binding to the host cell surface [28]. SARS-CoV-2 incorporates a polybasic cleavage site which is known to be responsible for increased transmissibility and pathogenicity in other viruses—for instance, avian IAV. In addition, SARS-CoV-2 possesses hemagglutinin esterase glycoprotein, which is used as an invading mechanism.

The influenza A virus particle is surrounded by a lipid bilayer which contains two main glycoproteins—hemagglutinin (HA) and neuraminidase (NA)—as well as the ion channel M2 protein. The inner surface envelops the M1 protein and the nuclear export protein (NEP) is located under the viral lipid bilayer. A complex made up of three polymerase subunits, PB2, PB1, and PA, is known as the viral RNA-dependent RNA polymerase [22].

In contrast, the virion structure of non-enveloped human rhinovirus is simpler [25]; its capsid is not covered by a lipid envelope [29] and is comprised of four viral structural proteins—VP1, VP2, VP3, and VP4. VP1 is the largest and most surface-exposed protein of the virus capsid. The remaining viral proteins are responsible for viral replication and subsequent assembly [18]. The virus capsid proteins exhibit a high degree of heterogeneity, resulting in a wide antigenic diversity [14,29]. Several antigenic sites have been identified for HRVs through studying the binding of neutralizing antibodies. Three distinct species of human rhinoviruses are currently known: HRV-A, HRV-B, and HRV-C. In total, over 170 types of HRV-A, HRV-B, and HRV-C were identified to be in circulation [30].

## 5. Physicochemical Properties

### 5.1. Environment

When discussing enveloped vs. non-enveloped viruses, the first thing that must be addressed is the outer protective covering surrounding enveloped viruses. However, being lipid-enveloped viruses, IAV and SARS-CoV-2 are environmentally labile to temperature (heat-sensitive) [42,43,44,45]; they are also disrupted by acids, detergents, and drying [42,43,44,45,46,47,48,49,50]. Viral particles are sensitive to oxidizing agents, lipid solvents, UV irradiation, and formaldehyde [48]. In contrast, non-enveloped rhinoviruses are environmentally more stable in elevated temperatures—they tolerate hot temperatures, displaying excellent heat-resistant properties, and can easily withstand a dry and acidic environment [51,52,53,54,55]. Compared to enveloped viruses, non-enveloped viruses are generally more powerful and proliferate rapidly in an acidic environment [56,57,58,59]. However, in contrast to many other non-enveloped picornaviruses [56,57], HRVs are acid-sensitive [2,34,56] and unstable below pH 5–6 [33,56] (Table 1).

### 5.2. Stability

Due to their complex outer structure, enveloped viruses including IAV and SARS-CoV-2 tend to show a higher stability and survive longer due to their adaptability to different environmental conditions [49,60]. In contrast, due to their virion structure and low resistance to the harsh environment, rhinoviruses are less stable than enveloped viruses and, therefore, survive for a shorter time in the host as well as outside the host environment [2,56].

### 5.3. Sterilization

Rhinoviruses, as with any non-enveloped viruses, are difficult to sterilize, as they can easily adjust to changes in temperature [56]. Enveloped viruses, on the other hand, are easier to sterilize because they do not show a high resistance to desiccation and heat treatment [53].

## 6. Genome Organization

There are differences in the genomic organization of SARS-CoV-2, HRV, and IAV. One out of the three viruses discussed, IAV, contains a negative genome, (–)RNA; two others possess positive-sense viral RNA, (+)RNA. All of them are single-stranded RNA viruses of a linear type of nucleic acid molecule. IAV contains segmented RNA and its genome consists of eight segmented genes of varying lengths: PB2, PB1, PA, HA, NP, NA, M, and NS. Each viral RNA genome segment encodes at least one protein [26] and is associated with a protein complex [22,27]. In contrast, the genomes of both SARS-CoV-2 and HRV are nonsegmented.

## 7. Viral Replication

The main stages of the viral replication cycle of IAV, HRV, and SARS-CoV-2 are presented in Table 1.

Viruses have specific receptors on the host cells that they attach to, and, in turn, each virus has a particular viral protein that binds the cell attachment receptor. All three viruses hijack the cell via endosomes [2,29,37,39]. SARS-CoV-2 can also enter cells via plasma membrane fusion [36].

The major surface protein of the HRV capsid, VP1, plays an important role in the attachment to the surface of a host cell [2,29,37]. After binding and endocytosis in the cell, single-stranded positive polar RNA migrates across the endosomal membrane into the cytoplasm. All stages of HRV replication, including virion assembly, occur in the host cell’s cytoplasm [35]. In the late stage of infection, mature virions are released from the cell by lysis or via non-lytic release [35].

SARS-CoV-2 attaches to host cells using the cell surface protein ACE2 (angiotensin-converting enzyme 2) through the receptor-binding domain of the spike (S) protein [37,41] which mediates SARS-CoV-2 entry into cells [36]. Moreover, similar to certain enveloped viruses, SARS-CoV-2 possesses a glycoprotein hemagglutinin, esterase, which is involved in the virus invading and attaching. The coronavirus genomic RNA encodes structural, nonstructural, and accessory proteins [61]. Nonstructural proteins play key roles in viral RNA synthesis, while structural proteins are important for virion assembly. SARS-CoV-2 mature virions leave the infected cell through the mechanism of exocytosis. The accessory proteins are not directly involved in viral replication but interfere with the host’s innate immune response [62,63].

Individual spikes on the virion of the IAV that attach/bind to the cell receptor—sialic acid—are molecules of the hemagglutinin. Attachment to the host cell occurs through the receptor-binding domain, which is located in the HA1 [39]. IAV entry also involves the HA and occurs through receptor-mediated endocytosis. NA is responsible for the release of mature viral particles from infected host cells. M1 protein is involved in virion assembly and budding, and the nuclear export protein NEP plays a role in the nuclear export of viral ribonucleoprotein complexes. The core of the IAV is made up of the eight viral RNA segments (genes) that are encapsidated by the viral nucleoprotein. Each ribonucleoprotein (RNP) complex is associated with a viral RNA-dependent RNA polymerase complex made up of the three polymerase subunits PB2, PB1, and PA, which, together with the viral NP, are the essential proteins required for viral replication and transcription. The multipotent enzymes of negative-strand RNA viruses, RNA-dependent RNA polymerases, transcribe and replicate the viral genome in the host [22]. Importantly, in contrast to HRV and SARS-CoV-2, virion RNA synthesis occurs in the nucleus.

Host cells do not normally replicate RNA; thus, RNA viruses must encode an enzyme, RNA polymerase (RNA-dependent RNA polymerase), which is required for genome replication and the production of mRNA. RNA-dependent RNA polymerase is an essential protein encoded in the genomes of all RNA-containing viruses with no DNA stage [64,65], including SARS-CoV-2 [66].

For all (−)RNA viruses, including IAV, the most important event in infection is the synthesis of mRNAs from the negative-sense genomic RNA by the RNA-dependent RNA polymerase. The mRNAs are translated into a single protein or more than one product. The naked genomes of (−)RNA viruses are not infectious, nor are they complementary RNA copies of the genomes [66,67].

For viruses with a positive-sense genome, following virus entry into the host cell the first step in replication is the translation of the incoming genomic (+)RNA, mRNA, on cytoplasmic ribosomes to produce proteins which are required for the synthesis of antigenomic copies of the genomic (+)RNA. As the replication cycle of (+)RNA viruses begins in the translation of the (+)RNA genome in order to produce RNA synthesis enzymes, the naked RNA is infectious. In other words, the introduction of the genomic RNA into a susceptible cell will result in a complete infection cycle [66,68]. New virions form by budding, thereby incorporating matrix proteins and viral RNPs that align below the plasma membrane regions containing viral envelope proteins.

New viral particles are released from the infected cell through budding (IAV), exocytosis (SARS-CoV-2), or lytic or non-lytic mechanisms (HRVs).

## 8. Pathogenesis

### 8.1. Temperature Sensitivity of Replication

As temperature-sensitive pathogens, human rhinoviruses have a relatively low optimum temperature for replication in HeLa cell culture, 33 °C, which reflects their adaptation for replication in the nasopharyngeal region [2,69]. However, some HRVs are temperature-resistant and may multiply at a higher temperature of 37 °C [52,70]. In MDCK cells, typical influenza A viruses may replicate in a wide range of temperatures from 32 to 38 °C [71]. In eggs, the cut-off temperature of IAVs is even higher and reaches 40 °C [72,73]. SARS-CoV-2 can also efficiently replicate in Vero cells in the range of 33–37 °C [74]. The ability of respiratory viruses to replicate in vitro out of a temperature optimum confirms the fact that these viruses can also infect the lower respiratory tract, thus causing its damage (Table 2).

### 8.2. Differences and Similarities between Influenza and COVID-19

The influenza virus and SARS-CoV-2 cause an acute respiratory infection that can manifest itself in a variety of ways, from asymptomatic to severe and even fatal. There are several differences between SARS-CoV-2 and influenza A virus pathogenesis. For instance, the IAV has a shorter incubation period and a shorter interval between subsequent infections. It is about 1–4 days, while in the case of a disease caused by SARS-CoV-2, it is up to 14 days. Although the symptoms of both diseases are similar, the proportion of patients with severe disease is different.

**Table 2:** Comparative characteristics of the common cold, influenza, and COVID-19: pathogenesis.

Properties [refs]	Causative Agent		
	Rhinovirus	Influenza Virus	SARS-CoV-2
PATHOGENESIS AND CLINICAL FEATURES [1,2,3,4,21,52,69,70,71,72,73,74,75,76,77,78,79,80,81,82,83,84,85,86,87,88,89,90,91,92,93,94,95,96,97,98,99,100,101,102,103,104]			
“Entrance gate” of infection (the primary route of entry)	Upper respiratory tract (mouth and nose)	Upper respiratory tract (mouth and nose)	Upper respiratory tract (mouth and nose)
Upper respiratory tract infection	Common	Common	Common
Replication at high temperature	Sometimes	Common	Common
Lower respiratory tract infection	Sometimes	Sometimes	Common
Incubation period	1–3 days	1–4 days	2–14 days
Symptoms	Gradual	Abrupt	Gradual
Sudden onset	Smooth onset	Very common early symptom	Sudden or smooth onset
Running nose (rhinorrhea)	Common	Common	Common
Nasal discharge	Common	Common	Common
Nasal congestion	Common	Sometimes	Common
Sneezing	Very common	Sometimes	Common
Loss of smell and taste	Rare	Sometimes	Very common early symptom
Shortness of breath	Mild	Sometimes	Common
Sore throat	Very common	Sometimes	Sometimes/common
Cough	Common (mild to moderate, hacking)	Common (dry cough, can be severe)	Common (dry cough, can be severe)
Headache	Rare	Common	Common
Muscle pain (body aches)	Sometimes (slight)	Very common (often severe)	Very common (often severe)
Chilliness and fever	Rare in adults, possible in children	Very common; may have chills	Very common; may have chills
Malaise	Sometimes	Very common	Very common
Fatigue, weakness	Sometimes	Very common (can last for weeks)	Very common (can last for weeks)
Diarrhea	Rare	Sometimes	Sometimes
Loss of appetite	Sometimes	Common	Sometimes

The clinical picture of COVID-19 is similar to that of influenza and many other acute viral respiratory infections: fatigue, breathing difficulties, cough or sore throat, fever, muscle pain, headache, nausea, insomnia, diarrhea, vomiting, etc. (see Section 9 below). A hallmark event is increasing shortness of breath, which may indicate the development of pneumonia. Complications from severe COVID-19 include the following: pneumonia, sepsis and septic shock, acute respiratory distress syndrome, heart injury, acute kidney injury, meningoencephalitis, myocarditis, gastrointestinal problems (especially in children), thrombosis, and renal failure [92].

COVID-19 can also cause neurological complications [93]. Most people with severe COVID-19 infection appear to recover without experiencing significant mental illness. Delirium may feature in the acute stages of COVID-19. Unfortunately, little is known about the pathogenesis of SARS-CoV-2 infection in the central nervous system. Kumari et al. [94] suggested that the direct infection of cells of the central nervous system together with the inflammatory response induced in the brain result in severe disease. As for influenza, the proportion of severe and extremely severe cases and complications is lower [95,96,97].

### 8.3. Pathogenesis of Rhinovirus Infection

The primary route of entry for HRVs, IAVs, and SARS-CoV-2 is the upper respiratory tract (mouth and nose). Rhinovirus infection of the nasopharyngeal mucosa does not cause symptoms of acute viral respiratory infection directly, instead initiating an inflammatory response that produces the symptoms [3]. Some inflammatory pathways, including neurologic reflexes triggered by the infection, have been identified as playing key roles in the pathogenesis of rhinovirus infection [98].

Generally, rhinovirus infection is mild and self-limiting, but it may also be associated with bronchiolitis (in infants), pneumonia (in the immunocompromised), and exacerbation (in patients with pulmonary conditions) [87]. HRVs are the major pediatric pathogens that affect both the upper and lower respiratory tracts and frequently cause wheezing, asthma exacerbations, and pneumonia [4,70]. A defective immune response to rhinovirus infection involving interferon-lambda is considered as one of the mechanisms behind exacerbations in asthmatic children [4].

Unlike the influenza virus, human rhinovirus does not cause cytopathic changes in the nasal epithelial cells [99]. However, it can cause cytopathology in the bronchial epithelium [70] and disruption in the epithelial barrier of the airway [100,101].

## 9. Clinical Manifestations

In general, the respiratory symptoms are typical for the majority of upper respiratory viral infections [1] and about the same for all viral pathogens. Studies on the symptoms specific to different respiratory viruses have indicated that it is not possible to identify the exact causative virus based on the symptoms alone, since similar symptoms are caused by different viruses [88]. It is difficult to define respiratory symptoms exactly because of the great variation in their severity, duration, and type [1]. With regard to particulars, COVID-19 is closer to influenza than to other upper respiratory viral infections due to the presence and severity of respiratory symptoms. There is much overlap in the symptomatology of common cold and influenza symptoms. A common early symptom of influenza is sudden onset [85,86]; other acute viral respiratory infections are characterized by a smoother onset, with loss of taste and smell as early distinguishing symptoms of COVID-19 [84].

The cold syndrome, including the infection caused by rhinovirus, has been defined as a short mild illness with early symptoms of sneezing, headache, stuffy nose, chilliness, and sore throat, as well as later symptoms of nasal obstruction, nasal discharge, body aches, possibly cough, and malaise [102]. Generally, the severity of symptoms increases rapidly, peaking 2–3 days after infection, with a mean duration of symptoms of 7–10 days.

In contrast, the most common symptoms of COVID-19 include headache, fever, loss of smell and taste, cough, nasal obstruction, asthenia, myalgia, rhinorrhea, gustatory dysfunction, shortness of breath, sore throat, body aches, fatigue, possibly diarrhea, and vomiting [89,103] (Table 2). There is some evidence to suggest that patients who have suffered from mild or severe COVID-19 can experience prolonged symptoms or develop long-term complications [103]. The risk of severe disease and death is higher in people who are older, male, and from deprived areas. Children and infants generally appear to experience milder symptoms than adults [103].

Influenza syndrome is typically characterized by fever, myalgia, headache, cough, nasal congestion, sore throat, loss of appetite, and weakness [1].

## 10. Epidemiology

### 10.1. Origin

The source of the COVID-19 outbreak has yet to be determined. It is suggested that COVID-19 likely has a zoonotic origin [105,106,107]. Alternatively, a human-made origin of SARS-CoV-2 has also been discussed. The influenza A virus causes human disease through strains that appear through the seasonal variation of human influenza viruses and through pandemic infection resulting from viral reassortment or the adaptation of zoonotic influenza viruses, thus introducing antigenically novel variants into the human population [108]. Rhinoviruses seem likely not to have arisen from a recent zoonotic event [109,110].

### 10.2. Transmission

HRV, IAV, and SARS-CoV-2 are respiratory pathogens that are transmitted by classical airborne droplets.

The person-to-person transmission of SARS-CoV-2 has occurred extensively. The transmission risk is the highest if people are in a close distance of within 2 m. The human-to-human transmission of novel coronaviruses mainly occurs through the spread of droplets or direct contact from the sneezing or coughing of an infected individual [82,103]. In addition to respiratory secretions, SARS-CoV-2 has been detected in feces, blood, and urine.

Yan et al. [111] proved that the influenza virus likely spreads by aerosols, not just coughs or sneezes. People infected with influenza can pass the virus to others just by breathing, with the role of transmission from coughing and sneezing smaller than previously thought. The transmission of the influenza A virus occurs mainly before symptoms appear, and for SARS-CoV-2 infection so-called “asymptomatic transmission” is possible.

The transmission of rhinovirus occurs by small- and large-particle aerosols or through direct contact [112]. To cause infection, the virus must be deposited on the nasal mucosa or conjunctiva; some believe that oral inoculation is not sufficient [3,51,113]. Feasible mechanisms for rhinovirus transmission are also hand-to-hand and hand-to-surface-to-hand transfer. Additionally, self-inoculation appears to be an effective means of rhinovirus transmission in the home environment. HRVs can remain viable for a few hours or up to 4 days on suitable surfaces [51,98,114]. This finding supports the possibility of self-inoculation by fomites.

### 10.3. Seasonality

SARS-CoV-2, much like influenza, tends to be a winter virus that feels more comfortable in cold and dry air [115]. However, so far, such conclusions should be drawn with caution. We know too little about whether the COVID-19 coronavirus is seasonal or not (Table 3).

HRV infections occur throughout all seasons but frequently peak in autumn and spring [87,98,116,117,118]. Their infection rate varies throughout the year due to the prevalence of other more seasonal respiratory viruses. However, epidemiologic data seem to suggest that the relative number of rhinovirus infections occurring during the year is fairly constant [98].

### 10.4. Morbidity, Hospitalization, and Mortality

The impact of respiratory virus infections on morbidity, hospitalization, and mortality in patients has been widely studied. In this review, we will restrict ourselves to the most general, global data. All three infections—influenza, the common cold, and COVID-19—are distributed worldwide. How do indicators of the severity of illness (incidence rate, hospitalization rate, and death rate) compare for these three infections?

The WHO estimates that, worldwide, annual influenza epidemics result in about one billion cases of infection [119], about 9,460,000 hospitalizations with severe illness [120], and about 410,000 deaths [121]. This estimate excludes the global incidence of influenza and deaths during the COVID-19 pandemic.

The global incidence of COVID-19 is different. The annual death rate from the COVID-19 results is about 2.8–6.2 times higher than that of seasonal influenza (ratios were calculated based on data from [120,121,122]). The rate of annual infection with SARS-CoV-2 is about 83,560,000 and the hospitalization rate is about 15,800,000 cases.

**Table 3:** Comparative characteristics of the common cold, influenza, and COVID-19: epidemiology.

Properties [refs]	Causative Agent		
	Rhinovirus	Influenza Virus	SARS-CoV-2
EPIDEMIOLOGY [1,10,11,82,84,86,88,89,90,120,123,124,125,126,127,128,129,130,131]			
Origin	Likely not arisen from a recent zoonotic event	Likely not arisen from a recent zoonotic event except pandemic strains	No conclusive evidence yet. Likely zoonotic origin
Transmission	Mainly through direct contact with aerosolized particles from an infected individual	Through the spread of droplets or direct contact by sneezing or coughing from an infected individual	Through the spread of droplets or direct contact by sneezing or coughing from an infected individual
World distribution	Worldwide	Worldwide	Worldwide
Seasonality (circulation pattern)	Cold seasons, but is possible year-round	Cold seasons (fall and winter)	No conclusive evidence yet. Possibly cold seasons
Level of transmissibility	Moderate	High	High
Annually infected (global), thds	No global data available	~1,000,000	~83,600
Hospitalization rate (global), thds	No global data available	~9460	~15,800
Annual death (global), thds	No global data available	~410	~1800
Prevalence and incidence of the disease	Local outbreaks	Epidemics, pandemics	Pandemic
Circulation during an influenza epidemic season	Does not change	Circulation of seasonal influenza viruses	Yes
Circulation during an influenza pandemic	Does not change	Decreased circulation of seasonal influenza viruses	Not applicable
Circulation during the COVID-19 pandemic	Does not change	Decreased circulation of seasonal influenza viruses	Yes

Despite HRV being the most common cause of acute viral respiratory infections and it being found year-round, the global burden of the disease and the global case count attributable to rhinovirus are unknown, most likely because rhinovirus surveillance is not yet well established. This may be due to the relatively mild course of rhinovirus infection. However, country-specific information is available for some countries. For instance, according to the Pan American Health Organization (PAHO), there are about 16,000 laboratory-confirmed rhinovirus infection cases in North America (average data from 2017 to 2020), with an average of 695 cases in Central America over the same period, and 185 rhinovirus cases in South America [132]. No statistics on hospitalization and mortality are available.

### 10.5. Prevalence and Incidence of the Disease

In contrast to HRVs, which cause local outbreaks only, type A influenza is the cause of epidemics and pandemics. As for COVID-19, scientists around the world are discussing the important question of whether SARS-CoV-2 will become a seasonal virus [133,134,135]. The majority of experts are sure that COVID-19 may eventually become a seasonal illness similar to influenza, but the virus will continue to spread year-round until the entire population achieves herd immunity.

### 10.6. Circulation

Novel coronavirus SARS-CoV-2 spread throughout the world very quickly. On 31 December 2019, the WHO China Country Office was first informed of a cluster of cases of pneumonia of unknown cause detected in China [103]. As of 20 July 2021, globally there have been 190,770,507 confirmed cases of COVID-19, including 4,095,924 deaths, reported to the WHO [122]. According to the information of the WHO [122] and Johns Hopkins University, today over 190 countries have reported cases of COVID-19 infection [136].

The common cold is an illness that frequently affects humans of all ages [98]. For most of the year, the most frequent human acute viral respiratory infections across all age groups are associated with HRVs [137]. Rhinoviruses disproportionately affect young children [98]. Adults usually catch colds from children [98]. Interestingly, adults who live with young children experience more colds than other adults living without young children [3]. The continuous invasion and high diversity of rhinoviruses have been observed [138]. Rhinovirus activity has been detected in countries of all continents: Spain [139], Brazil [140,141,142], Argentina [143], France [125], Italy [10,144], the United Kingdom [116], Russia [145], Finland [146,147], Canada [148], the US [3,102,137,149,150,151], Australia [152], Vietnam [153], China [154,155], Hong Kong [156], Japan [157], Burundi [158], KwaZulu-Natal (a province of South Africa) [159], Tanzania [90], and many others, including Antarctica [160].

Seasonal human influenza viruses circulate in all parts of the world. They cause a year-round disease burden [161].

## 11. Interactions between Rhinoviruses and Other Respiratory Viruses during Their Co-Circulation

As was mentioned above, the emergence of the recent 2019 novel coronavirus was associated with substantial reductions in the circulation of seasonal respiratory viruses. Pool et al. [162] reported that, before 2020, the most frequently detected non-COVID-19 virus was influenza, followed by rhinovirus. During the first wave of the 2020 pandemic, a significant drop in the circulation of non-COVID-19 respiratory viruses compared to previous years was detected; among them, the most common non-SARS-CoV-2 virus detected was HRV.

**Figure 2:**
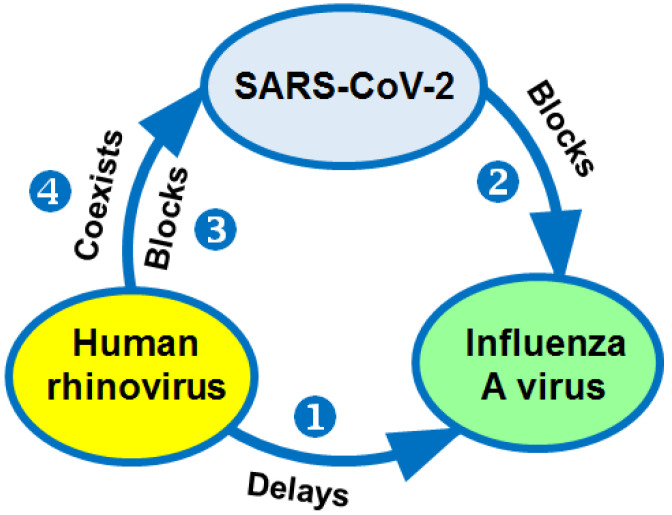
Interactions between human rhinoviruses, influenza A virus, and SARS-CoV-2 (based on the results and suggestions of [10,126,128,163,165]).

The possible impact of rhinoviruses on some other respiratory pathogens has been highlighted in the literature [125,126,127,130,163]. It has been shown that HRVs can both coexist with other respiratory viruses and compete with them. The mutual effects between HRVs, influenza viruses, and SARS-CoV-2 during their co-circulation manifest in several scenarios: (i) the spread of influenza viruses is delayed by seasonal HRVs [10,123,125,163,164] or (ii) by the SARS-CoV-2 pandemic [13]; in turn, pandemic SARS-CoV-2 (iii) can be blocked by HRVs [126] or (iv) co-circulate with them [10] (Figure 2).

### 11.1. The Spread of the Influenza A Virus Might Be Interrupted by HRV

A nine-year analysis of 44,230 cases of respiratory illness in Glasgow, UK, revealed that interplay between a ubiquitous human rhinovirus during its peak activity and seasonal influenza A virus can diminish the activity of the latter [166].

Some believed that interference between rhinovirus and the H1N1pmd09 virus disrupted the pandemic in Europe. Data from a number of European countries pointed out that the circulation of the H1N1pdm09 influenza A pandemic virus might be interrupted by the annual autumn rhinovirus epidemic [128,165]. Other studies have also demonstrated that the annual rhinovirus delayed the spread of the novel pandemic H1N1pdm09 influenza virus [123,163]. The researchers explained this phenomenon by the fact that infection with rhinovirus may inhibit subsequent influenza infection by activating antiviral defenses in the airway mucosa, which is a target tissue of both viruses. These results confirmed that viral interference could affect the course of an influenza epidemic or even pandemic.

As we mentioned above, rhinoviruses have long been known as one of the most frequent causes of colds. Moreover, they are also present at high rates in asymptomatic people. Moreover, such asymptomatic infections can trigger interferon-stimulated gene expression in the upper respiratory epithelium [167,168]. Thus, rhinovirus infections might protect the host by blocking infection by viruses of higher virulence.

### 11.2. Influenza Viruses Are Delayed in Spread by SARS-CoV-2

The world has experienced five viral pandemics in the last hundred years; in particular, four pandemics occurred due to influenza A viruses and the last one occurred due to SARS-CoV-2. In each known influenza pandemic, the causative pandemic strain has replaced the previous seasonal virus. According to the latest update of the WHO, as of 5 July 2021 global influenza activity was at lower levels than expected for this time of the year.

In the Northern Hemisphere, influenza activity returned to inter-seasonal levels, while in the Southern Hemisphere the influenza season has not started yet [13]. Most countries reported the sporadic detection of influenza to the WHO, though many areas are experiencing a high rate of COVID-19 cases [13]. In areas where COVID-19 is widespread, the circulation of influenza viruses is still significantly reduced.

Notably, throughout the pandemic caused by SARS-CoV-2, there has been a 99% reduction in influenza virus isolation globally [12]. The most common co-infections in the COVID-19 pandemic were HRV, HRSV, and non-SARS-CoV-2 coronaviruses. The lowest levels of detection of co-infection with SARS-CoV-2 were influenza and adenoviruses [11]. It is anticipated that influenza will re-emerge following the SARS-CoV-2 pandemic and circulate again [12].

### 11.3. HRV Van Block SARS-CoV-2

Competitive relationships have been registered not only for HRV+IAV but also for the HRV+SARS-CoV-2. According to the extraordinary study of the MRC-University of Glasgow Centre for Virus Research [126], HRV infection can block SARS-CoV-2 replication in cells of the respiratory tract by triggering an interferon response, and it may reduce COVID-19 severity and disease burden.

### 11.4. SARS-CoV-2 Can Co-Circulate with Other Respiratory Pathogens

A study performed in Italy during the wintertime between December 2019 and March 2020 showed an interesting trend in the dissemination of respiratory pathogens; HRV (22.3%) and HRSV (23.7%) were found to be the most commonly identified viruses, followed by SARS-CoV-2 (14.1%) [10].

Wu et al. [128] suggested that the annual rhinovirus outbreaks may determine the timing and severity of seasonal influenza epidemics and the ongoing COVID-19 pandemic. These data indicate the necessity of an update of the current conception of rhinovirus infection. The tragic events of 2019–2021 highlight the necessity of understanding and predicting the circulation of ubiquitous respiratory viruses during the COVID-19 pandemic to design effective anti-pandemic measures.

## 12. A Brief Summary of the Main Similarities and Differences between SARS-CoV-2, IAV, and HRV

Human rhinoviruses are distributed globally and cause acute viral respiratory infections across all age groups [118]. They have long been considered relatively harmless viruses and have been undeservedly neglected in the past because they were generally perceived as respiratory viruses only capable of causing a mild common cold [100]. Of course, the statement that HRVs are one of the most common causes of mild respiratory infections is correct. However, they also induce aggravations of asthma; chronic obstructive pulmonary disease; bronchiolitis in young children; and infections of the lower respiratory tract in children, the elderly, and immunosuppressed patients. Antigenic diversity and a large number of HRVs (currently over 170) are significant obstacles in rhinovirus vaccine development [169].

Moreover, in the light of recent data, it became known that rhinoviruses may not only coexist in parallel with other respiratory viruses but also compete with them (Figure 2). This suggests that the role of rhinoviruses in the epidemiological process is underestimated and that they need to be comprehensively studied. Until now, it has not been clear as to why rhinoviruses continue to circulate and even delay the spread of SARS-CoV-2 [126] or H1N1pdm09 viruses [123,125,163,164], while seasonal influenza viruses are being supplanted by the novel pandemic coronavirus [13,130].

How distant or similar is the human rhinovirus to the influenza A virus and SARS-CoV-2? In Table 1, Table 2 and Table 3, we can see how many features distinguish rhinoviruses from influenza viruses. IAV and SARS-CoV-2 are known to be viruses of medium size. Their capsid is covered by a lipid envelope. The influenza A virus contains negative-sense single-stranded segmented RNA, classified within the *Orthomyxoviridae.* The flu A virus has a small genome (~14 kb) encoded in eight strands of RNA, and it infects human cells differently than coronaviruses [170]. Non-enveloped human rhinoviruses are a small-sized pathogen that possess a positive-sense single-stranded RNA (~7 kb) and are classified within the *Picornaviridae*.

SARS-CoV-2 possesses positive-sense viral nonsegmented RNA; it is a member of the family *Coronaviridae.* Interestingly, coronaviruses have the largest genomes of any known RNA viruses (~30 kb). These large genomes have led some to suspect the presence of a so-called “proofreading mechanism” to reduce the mutation rate and stabilize the viral genome. Indeed, coronaviruses have a proofreading exonuclease ExoN, which explains their relatively low mutation rates (~10^–6^ per site per cycle) if compared to the influenza virus (~3 × 10^–5^ per site per cycle) [171]. Bar-On et al. [170] suggested that “This relatively low mutation rate will be of interest for future studies predicting the speed with which novel coronaviruses can evade our immunization efforts” [170].

HRV, IAV, and SARS-CoV-2 have many biological properties in common: they are found worldwide and are the most frequent RNA respiratory viruses associated with globally distributed respiratory infections. Alternatively, apart from the dissimilarities in their structure, these viruses exhibit different biological characteristics too, although their differences in clinical presentation are not so pronounced that we can identify the causative agent of the acute respiratory infection exactly. Although the respiratory symptoms of both diseases are similar, the proportion of patients with severe disease is higher for patients infected with SARS-CoV-2. The influenza A virus has a shorter incubation period than SARS-CoV-2.

Seasonal variations with predominance during cold seasons are typical for rhinovirus infections [116,141]. Influenza viruses circulate in the winter season [172]. Regarding SARS-CoV-2, it is too early to talk about the seasonality of its distribution. Six months after the beginning of the COVID-19 pandemic, the WHO suggested that the new coronavirus is not seasonal, differing from other coronaviruses, and does not occur in waves. “It’s going to be one big wave,” WHO spokesperson Dr. Margaret Harris said during a press conference in July 2020 [173]. The position of the WHO was that COVID-19 is not affected by cold temperatures in a manner similar to seasonal influenza nor by heat, and there is no evidence that COVID-19 will follow seasonal variations. It has been suggested that season does affect the transmission of the virus because a large number of COVID-19 cases are associated with cold and dry climates in temperate regions of the world. Thus, the seasonality of the SARS-CoV-2 spread is suspected [174]; there are concerns that SARS-CoV-2 may establish itself as an endemic human respiratory coronavirus [175].

Interestingly, in the pathogenesis of rhinovirus infection, there is a very important point that distinguishes rhinoviruses from other respiratory pathogens and brings them closer to coronaviruses. In contrast to many other respiratory viruses, the clinical symptoms of HRV infection are primarily caused by the host’s immune response to infection rather than by viral cytopathicity [176]. Intriguingly, whether during COVID-19 or influenza pandemics, HRVs are the most common viruses detected in patients meeting the appropriate clinical criteria for presentation to hospitals or clinics. Rhinoviruses, therefore, create an enormous socio-economic burden across the globe [110,177]. Maybe this is an answer to many of the questions raised.

It is speculated that any of the properties listed in Table 1, Table 2 and Table 3 or their combinations may somehow affect the uniqueness of the epidemiological process during rhinovirus infection in the COVID-19 era. Which of these are key points, individually or collectively, and whether they are is not known to us.

Not everything is so simple—for instance, this scheme does not fit HRSV, which is known to circulate at approximately the same intensity as rhinoviruses and does not disappear during COVID-19 or influenza pandemics. At the same time, there is little in common between such phylogenetically distant viruses as HRSV and rhinoviruses. HRSV is a member of the phylum *Negarnaviricota*, together with the influenza A virus [33]. By virion organization, this medium-sized virus which possesses a negative-sense RNA genome [32,33] is closer to the influenza virus. In terms of its structural and biological characteristics, there are far more differences than similarities between HRVs and HRSVs.

Thus, among all the viral characteristics presented above, there are no common global features uniting HRVs and HRSVs that could explain why their unique circulation is not affected by other viral pathogens. Phylogenetically and in terms of their main biological properties, they are very distant each from other. Their membership in the same kingdom of *Orthornavirae* is unlikely to play a key role. It is most likely that the reason for this lies in the yet-undiscovered subtle molecular mechanisms of infections caused by these agents.

## 13. Conclusions

“I know that I know nothing…”

*—Socratic paradox*

Many questions remain open: what allows some respiratory pathogens such as SARS-CoV-2 and HRV to coexist, while SARS-CoV-2 displaces influenza viruses and some others? What is the reason for the peculiar coexistence of the two distinct respiratory pathogens, the ubiquitous human rhinoviruses first discovered in the 1950s and the newly emerged SARS-CoV-2?

In nature, there are two different outcomes of competition between the two species: (i) competitive exclusion or (ii) coexistence. Competition is a situation where two different species can compete for the same abiotic resource. If competing species make use of the resources available in the environment in different ways, they can coexist in the same area. Can we attribute the first scenario to influenza viruses, adenoviruses, etc., and the second scenario to HRVs? There are still too many “white spots” for us to draw conclusions about this. Much about coronaviruses is still unclear.

What conclusions is it possible for us to draw at the moment? We know what the viruses we discussed in this review have in common and how they differ. We do know that the circulation of rhinoviruses is independent of the epidemiological process of other infections. However, we do not know why.

Collectively, new clinical findings and experimental insights relating to human rhinovirus should stimulate renewed interest in studying all common cold viruses, including rhinoviruses.

## Data Availability

Not applicable.
